# Dosimetric scorecards express precise clinical intent: alternate hippocampal-sparing whole-brain RapidPlan models favoring target coverage and homogeneity at 30 and 20 Gy

**DOI:** 10.3389/fonc.2024.1465171

**Published:** 2024-11-27

**Authors:** Kareem Rayn, Anthony Magliari, Ryan Clark, Lesley Rosa, Robert Doucet, Line Comeau, Alan Nichol, Russell Ruo, David Roberge

**Affiliations:** ^1^ Office of Medical Affairs, Varian, A Siemens Healthineers Company, Palo Alto, CA, United States; ^2^ Department of Radiation Oncology, Moffitt Cancer Center, Tampa, FL, United States; ^3^ Department of Radiology, Radiation Oncology and Nuclear Medicine, University of Montreal Health Centre, Montreal, QC, Canada; ^4^ Medical Physics Department, McGill University Health Centre, Montreal, QC, Canada; ^5^ Department of Surgery, BC Cancer, Vancouver, BC, Canada

**Keywords:** dosimetric scorecard, RapidPlan, autoplanning, hippocampal sparing, hypofractionation

## Abstract

**Introduction:**

This study develops two new multi-institutional hippocampal-sparing whole-brain RapidPlan™ models (HLS-EC-WB and HMS-EC-WB) inspired by CCTG-CE.7 featuring enhanced target coverage with varying hippocampal sparing (limited and moderate).

**Methods:**

New dosimetric scorecards were created to quantify the models’ clinical intent. The models were trained using a multi-institution dataset, and a recursive method was employed to generate consistent, high-quality plans. The models were validated using a five-case set and compared at 20- and 30-Gy prescriptions.

**Results:**

Each model scored highest on its associated dosimetric scorecard. The new models achieved higher brain PTV prescription coverage (98%–99%) compared to the previous HSWBv2 model (95.12%), with some trade-off in hippocampal sparing.

**Conclusions:**

Three high-quality automated RapidPlan™ models for hippocampal-sparing whole brain are now available, each with a distinct dosimetric scorecard. The new models prioritize increased PTV coverage at some expense to hippocampal sparing. All models, example plans, scorecards, and scoring tools are freely available online.

## Introduction

The NRG CC001 clinical trial demonstrated that limiting hippocampal radiation doses (D_min_ ≤ 9 Gy and D_max_ ≤ 16 Gy) when prescribing 30 Gy in 10 fractions of whole-brain radiotherapy preserved neurocognition better than traditional whole-brain radiotherapy for patients who had no metastases in, or near, the hippocampi. In 2022, the ASTRO clinical practice guidelines were updated to reflect this new standard of care (1). That same year, to better facilitate the automated planning of HSWB, a Version 2.0 RapidPlan™ model (HSWBv2) was released. This new HSWBv2 model follows the maximal hippocampal-sparing intent of NRG-CC001, utilized a complex recursive model training process, and was demonstrated to generate higher-quality hippocampal-avoidance whole-brain radiation therapy treatment plans through reductions in hippocampal dose while improving target coverage and dose conformity/homogeneity (2). These improvements were quantified in a new V2.0 dosimetric scorecard (2).

An ongoing phase III trial (CCTG-CE.7) aims to compare the overall survival and neurocognitive progression-free survival of stereotactic radiosurgery vs. hippocampal-avoidant whole-brain radiotherapy plus memantine in patients with five or more brain metastases (3). In contrast to NRG-CC001, patients with metastases in, or near, the hippocampi are eligible for the CCTG-CE.7 clinical trial, which prioritizes coverage of brain metastases when they are located in the hippocampal-avoidance volume (5 mm from the hippocampi) allowing for partial hippocampal sparing (3, 4). In many centers outside the USA, the standard prescription for whole-brain radiotherapy is 20 Gy in five fractions (5–7). This study aims to extend the HSWB approach by developing new models optimized for patients where brain metastases may be in, or near, the hippocampi and compatible with a prescription of 20 Gy in five fractions.

The 2022 HSWBv2 model favored hippocampal sparing over PTV coverage within NRG-CC001 protocol constraints. Alternative intents can be implemented such as a focus on target coverage or homogeneity. Here, we develop two new dosimetric scorecards to capture these alternative intents and use them to guide the creation of new highly tuned RapidPlan™ models to fully automate creating treatment plans, which best embody these specific intents. These two dosimetric scorecards and associated multi-institutional RapidPlan™ models are known as Hippocampal Limited-Sparing Enhanced-Coverage Whole-Brain 20 Gy (HLS-EC-WB) or “limited sparing model” and Hippocampal Moderate-Sparing Enhanced-Coverage Whole-Brain 30-Gy—scalable (HMS-EC-WB) or “moderate sparing model” both of which will also be compared against the standard intent of maximal hippocampal sparing of the 2022 HSWBv2 model and dosimetric scorecard.

## Materials and methods

### Scorecard tool

Dosimetric scorecards use established scoring methodology of multiple piecewise linear score functions to measure specific plan quality metrics (8). The PlanScoreCard Eclipse Scripting Application Programming Interface (ESAPI) tool, available free on the Varian Medical Affairs Applied Solutions (MAAS) GitHub, was used to create scoring metrics, automatically generate additional optimization and evaluation structures, and score candidate plans utilizing batch mode and CSV report output for easy data analysis (9). The normalize to max score feature of the MAAS-PlanScoreCard tool was utilized in this work, which reports scores after finding the dose re-normalization value, which results in the highest score. This allows each plan to be closely aligned to the physician’s actual intent, especially when exact target coverage goal is not stated, but instead, a range of acceptable target coverage is weighed relative to sparing goals. Normalize to max score reduces the noise when compared to scoring plans directly from dose optimization. Piecewise linear scoring functions slope steepness and relative scores, assigned to target coverage metrics, prevent automatic dose normalization outside the desired target coverage range.

### Dosimetric scorecards as a comprehensive articulation of a precise clinical intent

Two new dosimetric scorecards were created to precisely quantify the differences in clinical intent for both limited and moderate hippocampal sparing when compared to the existing dosimetric scorecard used with the 2022 HSWBv2 model (**Supplementary S1, S2**). Both dosimetric scorecards were created with physician guidelines to explicitly quantify the plan metric tradeoffs for each intent. A high-level overview of the differing intents can be seen in **Table 1**. A complete accounting of how points were assigned between the score metrics in each dosimetric scorecard can be seen in **Table 2**.

**Table 1:** 20Gy hippocampal limited sparing enhanced coverage whole brain was derived from an institutional protocol with less aggressive hippocampal sparing goals and thus cannot scale to 30Gy.

Intent	20Gy/5fx-Not scalable Limited Sparing Enhanced Coverage HLS-EC-WB	30Gy Scalable Rx Moderate Sparing Enhanced Coverage (HMS-EC-WB)	30Gy scalable Rx Aggressive Sparing HSWBv2
**Hippocampus Dmin**	7.6Gy (**38% Rx**) ALARA	9Gy (30% Rx) ALARA	9Gy (30% Rx) ALARA
**Hippocampus D0.03cc**	13Gy (**65% Rx**)	16Gy (53.3% Rx)	16Gy (53.3% Rx) **ALARA**
**PTV Rx** **dose coverage**	20Gy @ 98%-99%	30Gy @ 98%-99%	**30Gy @** >**95%**

Colors are included for readability. Bold or shaded areas highlight the differences from the moderate sparing model.

**Table 2:** Overview of the differences between the dosimetric scorecard point distribution for the various clinical intents each variation represents.

Id	StructureId	Metric Text	Max Score	Id	StructureId	Metric Text	Max Score	Id	StructureId	Metric Text	Max Score
0	PTV_2000	Volume at 20Gy [%]	20	0	PTV_3000	Volume at 30Gy [%]	**20**	0	PTV_3000	Volume at 30Gy [%]	**15**
1	PTV_2000	Dose at 98% [Gy]	14	1	PTV_3000	Dose at 98% [Gy]	14	1	PTV_3000	Dose at 98% [Gy]	14
2	PTV_2000	Dose at 95% [Gy]	2	2	PTV_3000	Dose at 95% [Gy]	**2**				
3	PTV_2000	Dose at 2% [Gy]	11	3	PTV_3000	Dose at 2% [Gy]	11	2	PTV_3000	Dose at 2% [Gy]	11
4	PTV_2000	Volume at 105% [%]	**5.5**	4	PTV_3000	Volume at 105% [%]	**5**	3	PTV_3000	Volume at 105% [%]	**5.5**
5	PTV_2000	Dose at 0.03CC [Gy]	**8**	5	PTV_3000	Dose at 0.03CC [Gy]	**2.5**	4	PTV_3000	Dose at 0.03CC [Gy]	**4.5**
6	PTV_2000	MaxDose [Gy]	**5**	6	PTV_3000	MaxDose [Gy]	**2.5**				
7	PTV_2000	HI [1 - 99]/20]	2	7	PTV_3000	HI [1 - 99]/30]	2	5	PTV_3000	HI [1 - 99]/30]	2
8	PTV_2000	Conformation No. at [12.7Gy]	1	8	PTV_3000	Conformation No. at [28.5Gy]	1	6	PTV_3000	Conformation No. at [28.5Gy]	1
9	Hippocampi	Dose at 0.03CC [Gy]	**7.5**	9	Hippocampi	Dose at 0.03CC [Gy]	**12**	7	Hippocampi	Dose at 0.03CC [Gy]	**7.5**
10	Hippocampi	MeanDose [Gy]	**12**	10	Hippocampi	MeanDose [Gy]	**16**	8	Hippocampi	MeanDose [Gy]	**12**
11	Hippocampi	Dose at 100% [Gy]	17	11	Hippocampi	Dose at 100% [Gy]	17	9	Hippocampi	Dose at 100% [Gy]	17
12	OpticChiasm	Dose at 0.03CC [Gy]	3.5	12	OpticChiasm	Dose at 0.03CC [Gy]	3.5	10	OpticChiasm	Dose at 0.03CC [Gy]	3.5
13	BrainStem	Dose at 0.03CC [Gy]	3	13	BrainStem	Dose at 0.03CC [Gy]	3	11	BrainStem	Dose at 0.03CC [Gy]	3
14	SpinalCord	Dose at 0.03CC [Gy]	3.5	14	SpinalCord	Dose at 0.03CC [Gy]	3.5	12	SpinalCord	Dose at 0.03CC [Gy]	3.5
15	OpticNerve_L	Dose at 0.03CC [Gy]	3.5	15	OpticNerve_L	Dose at 0.03CC [Gy]	3.5	13	OpticNerve_L	Dose at 0.03CC [Gy]	3.5
16	OpticNerve_R	Dose at 0.03CC [Gy]	3.5	16	OpticNerve_R	Dose at 0.03CC [Gy]	3.5	14	OpticNerve_R	Dose at 0.03CC [Gy]	3.5
17	Eye_L	MaxDose [Gy]	2	17	Eye_L	MaxDose [Gy]	**2**				
18	Eye_L	MeanDose [Gy]	2	18	Eye_L	MeanDose [Gy]	**2**	15	Eye_L	MeanDose [Gy]	**3.5**
19	Eye_R	MaxDose [Gy]	2	19	Eye_R	MaxDose [Gy]	**2**				
20	Eye_R	MeanDose [Gy]	2	20	Eye_R	MeanDose [Gy]	**2**	16	Eye_R	MeanDose [Gy]	**3.5**
21	LacrimalGland_L	MeanDose [Gy]	3.5	21	LacrimalGland_L	MeanDose [Gy]	3.5	17	LacrimalGland_L	MeanDose [Gy]	3.5
22	LacrimalGland_R	MeanDose [Gy]	3.5	22	LacrimalGland_R	MeanDose [Gy]	3.5	18	LacrimalGland_R	MeanDose [Gy]	3.5
23	Lens_L	Dose at 0.03CC [Gy]	2.25	23	Lens_L	Dose at 0.03CC [Gy]	2.25	19	Lens_L	Dose at 0.03CC [Gy]	2.25
24	Lens_R	Dose at 0.03CC [Gy]	2.25	24	Lens_R	Dose at 0.03CC [Gy]	2.25	20	Lens_R	Dose at 0.03CC [Gy]	2.25
25	_Brain&BODY	Volume at 99.5% [CC]	5	25	_Brain&BODY	Volume at 99.5% [CC]	5	21	_Brain&BODY	Volume at 99.5% [CC]	5
26	_Brain&BODY	MaxDose [%]	5	26	_Brain&BODY	MaxDose [%]	5	22	_Brain&BODY	MaxDose [%]	5
27	_BrainStem#Hi	Dose at 95% [Gy]	2	27	_BrainStem#Hi	Dose at 95% [Gy]	2	23	_BrainStem#Hi	Dose at 95% [Gy]	2
28	_Eyes&BODY	MeanDose [Gy]	5	28	_Eyes&BODY	MeanDose [Gy]	5	24	_Eyes&BODY	MeanDose [Gy]	5
**HLS-EC-WB Scorecard Total**			**158.5**	**HMS-EC-WB Scorecard Total**			**158.5**	**HSWBv2 Scorecard Total**			**142**

Hippocampal Limited Sparing (HLS-EC-WB) on the left has more points allocated to reducing hotspots in the target whereas Moderate Sparing (HMS-EC-WB) allocates those points to further hippocampal sparing. These can both be contrasted with the older 2022 scorecard with fewer metrics and total points. The above table doesn’t include the specific piecewise linear scoring function, the specific scoring value ranges where each metric fails (0 points), only where max points are awarded. The point distribution differences between neighboring scorecards are highlighted.

Colors are included for readability. Bold or shaded areas highlight the differences from the moderate sparing model.

**Figure 1:**
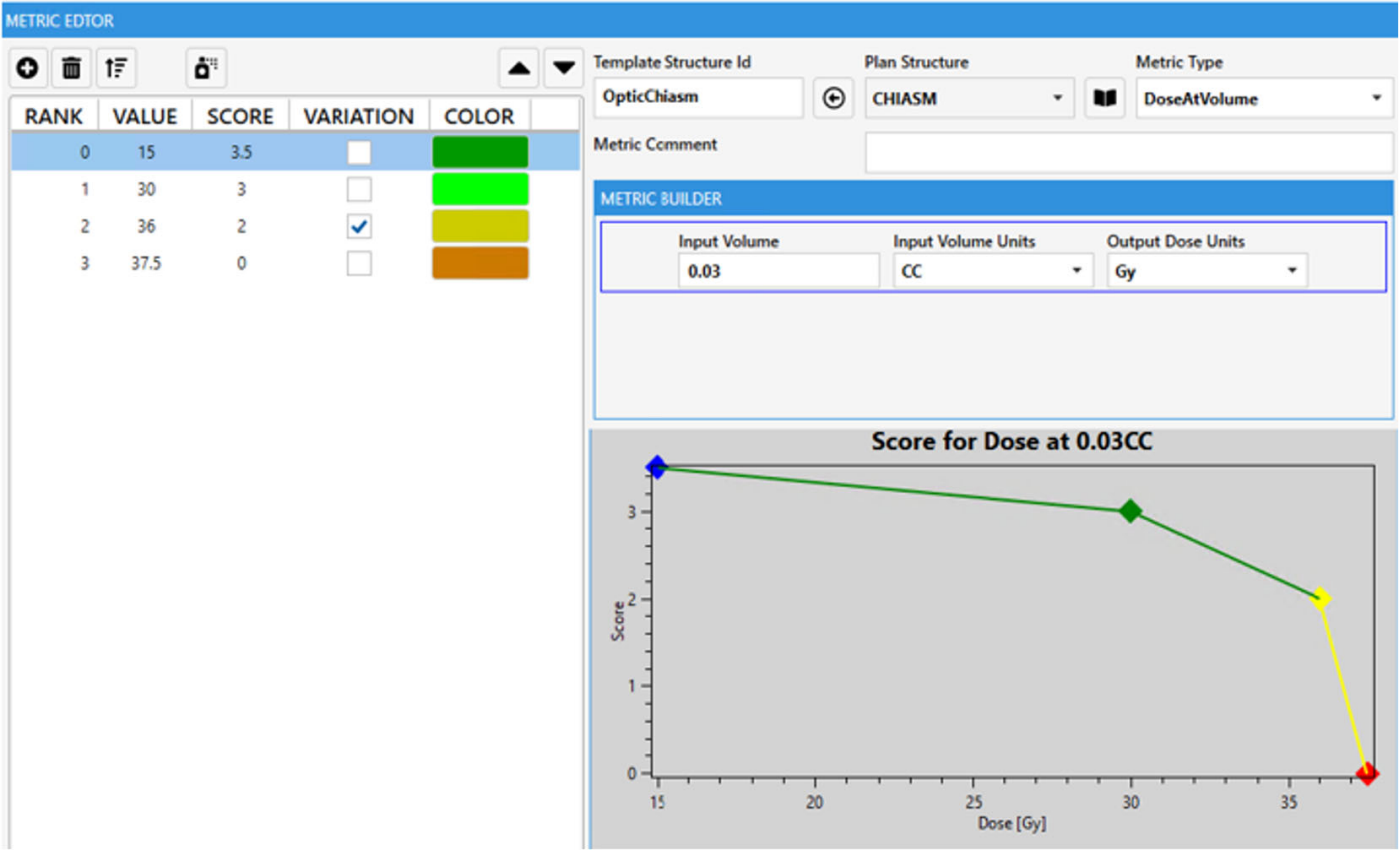
Example single scorecard metric for optic chiasm with piecewise linear scoring function plotted. A zero score represents a protocol violation, the steep sloping yellow section represents where a protocol may site a variation acceptable range. The green portion could be considered aspirational where ALARA principles can be relatively quantified for each OAR.

In general, how points are assigned between the various competing metrics represents the physician’s preference insofar as relative weighting. However, the view in **Table 2** omits the DVH value range each metric spans and the second-order priority encoded within each metric in the form of a piecewise linear function. Each function spans a range starting with the failing value (0 points) through the maximum, but often purposely unachievable, point value in each metric. Optional intermediate point values can be added in between to create the function shape and provide multiple levels of reasonable expected DVH values (**Figure 1**). Ideally, most maximum values are not achievable so as to continue to quantify additional improvement in already “very good” treatment plans. Care must be taken when attempting such a precise articulation of clinical intent. Some of the power of capturing such a comprehensive form of prescription at the protocol level will be demonstrated throughout this work. The most obvious advantage here is a singular objective measure of dosimetric plan quality per each specific intent from which the RapidPlan™ optimization objective tuning can be manually iterated upon. This laborious model tuning process can prove worthwhile when such a RapidPlan™ model is deployed in a clinic and works as a single-button press auto planning solution of high quality (as specifically defined by the associated dosimetric scorecard that RapidPlan™ model was tuned against).

### Model training overview

These new limited and moderate hippocampal sparing models were trained with the same final 42 case multi-institution CT datasets as HSWBv2 (2); structures were modified as needed (**Supplementary S5**). Each case was simulated with aquaplast mask immobilization and neutral head position.

For the limited sparing model, cases were initially re-planned to 20 Gy in five fractions, while for moderate sparing, cases were planned to 30 Gy in 10 fractions. All training set cases were created with 6X-FFF energy on a Varian Halcyon with SX2 MLC mode.

All cases utilized the VMAT technique. Four arcs had alternating clockwise and counterclockwise gantry rotations with collimator positions set at 315°, 0°, 45°, and 90°. The coplanar arcs had 359.8° of arc rotation and were positioned with the isocenter located in the center of the target.

The recursive method of model creation was utilized to generate a RapidPlan™ model with very consistent, high-quality plans developed with tight DVH prediction bands allowing for finely balanced hippocampal sparing, target coverage, and homogeneity optimization objectives to be used. Both the limited and moderate-sparing training set cases started by reoptimizing plans created from HSWBv2 with limited-sparing model leveraging the prescription (Rx) scaling feature (30 -> 20 Gy). No dosimetric or structure outliers occurred in either model as the 42 training set cases were the result of having already removed the anatomical outliers in the prior HSWBv2.

### Training hippocampal limited-sparing enhanced-coverage whole-brain 20 Gy

RapidPlan™ derives patient-specific DVH estimates for each OAR from a training set of treatment plans. This estimate has upper and lower bound predictions, which form a band. RapidPlan models can use line objectives, which are created near the lower portion of the DVH prediction band to drive the dose within the predicted range throughout that OAR’s volume. The starting point plans for the limited sparing model were created without the HSWBv2 model’s hippocampal DVH prediction line objectives as this cannot be altered, and instead, DVH upper point objectives were generated along the hippocampus lower bound prediction, which were then offset by fixed percentages toward higher dose levels. This offset accounted for the dose gradient shifting toward the hippocampal structures to achieve the desired target coverage goal (Rx dose covering 98%–99% of PTV_WB).

Those initial plans created from HSWBv2 model scaled to 20 Gy and, with offset hippocampal-sparing objectives, became the training set for the initial limited-sparing model. A recursive model creation process was employed to ensure the final limited-sparing training set consisted, exclusively, of plans generated from the initial limited-sparing model. Evaluating plan scores at each step in the process informed multiple iterations of re-tuning the optimization objective set priorities for continual improvement of the average score across the training set (**Supplementary S3**).

### Training hippocampal moderate-sparing enhanced-coverage whole-brain 30 Gy—scalable

The moderate-sparing model started from the same training set as the initial limited sparing model but with dose scaled to 30 Gy. Again, when replanning the training set cases, hippocampal DVH prediction line objective was not used. Instead, DVH point objectives were generated along the hippocampus line objectives but then offset by fixed percentages toward lower dose levels to make this moderate version of the model have more aggressive sparing of the hippocampus than the limited version (which was not designed to achieve 30-Gy protocol goals for a dose at 0.03 cc in the hippocampus). Plan scores on the moderate sparing scorecard were very good after reoptimizing the cases with manual fixed offset optimization objectives for the hippocampus.

No set of automatically generated optimization objectives or tuned priorities thereof could be found to create plans to outscore the previous cases when using those cases as a training set for an attempted final model. The line objective for the hippocampus was not aggressive enough. The solution was to perform another re-optimization of the training set cases this time purposely offsetting the hippocampus objectives by a fixed amount even lower than would create an optimal score, a so-called “sandbagging” re-optimization step. This created plans that overall scored worse but had more aggressive hippocampal sparing. Those cases were the training set for the final moderate sparing model, which could have optimization priorities tuned along a DVH line objective, which was sufficiently aggressive to finally generate plans scoring higher on the moderate-sparing scorecard then the first re-optimization set scored with manually generated fixed offset objective values (**Supplementary S4**).

### Model validation

Five separate cases not included in the training set were used for validation. All three distinct dosimetric scorecards were compared against all three model-produced plans at 20- and 30-Gy Rx. In addition to four arc Halcyon plans, both models were validated, with scores provided for multiple beam arrangements on TrueBeam. Additional validation of algorithm versions, beam energies, and more are outside the scope of this manuscript but can be seen in each RapidPlan™ model’s attached clinical description.

## Results

### Dosimetric summary table on HLS-EC-WB, HMS-EC-WB, and HSWBv2 on Halcyon: different tradeoffs

Treatment plans created from each model in a single-button press have their plan doses and associated scorecard doses scaled to the same Rx in **Table 3**. On five validation cases, average hippocampus mean and minimum (D100%) are close between both Enhanced-Coverage models with slight improvement seen with moderate sparing, whereas HSWBv2 demonstrates vastly improved mean and minimum Hippocampal dose. Between the Enhanced-Coverage models, the biggest difference is seen near the max (D0.03 cc) hippocampal dose where the moderate sparing model easily bests the 30-Gy protocol variation dose threshold of 16 Gy. The Limited-Sparing model was designed for an institution-specific 20-Gy prescription protocol and trades max hippocampal dose for slightly improved prescription dose homogeneity. Failing results for the Limited-Sparing model were included in the 30-Gy table for completeness but will not be reported further as that model is not designed to achieve 30-Gy dose constraints. However, the moderate sparing and HSWBv2 models can scale down to 20 Gy. Finally, Enhanced Coverage is reported for both new models near 99%, while HSWBv2 = 95.12%. Enhanced PTV coverage helps ensure any metastases in the vicinity (5–8 mm) of the hippocampus get prescription dose.

**Table 3:** Average key dose and score values from 5 validation cases with Halcyon treatment plans created from each model in a single button press having their plan doses and associated scorecard doses scaled to either 20Gy or 30Gy prescription.

Halcyon 4arc Validation Results Summary						
Rx: 20Gy				Rx: 30Gy		
Target/OAR	HLS-EC-WB	HMS-EC-WB	HSWBv2	Target/OAR	HMS-EC-WB	HSWBv2
Hippocampus				Hippocampus		
D100% (Gy)	4.84	4.76	3.79	D100% (Gy)	7.14	5.685
Dmean (Gy)	7.96	7.35	5.09	Dmean (Gy)	11.025	7.635
D0.03CC (Gy)	11.26	9.62	6.75	D0.03CC (Gy)	14.43	10.125
Brain PTV				Brain PTV		
V100% (Gy)	99.06	98.91	95.12	V100% (Gy)	98.91	95.12
D0.03cc (Gy)	21.9	22.16	22.05	D0.03cc (Gy)	33.24	33.075
V105 (%)	7.13	8.72	4.39	V105 (%)	8.72	4.39
Total score (%)	141.48/158.5 (89.28%)	136.29/158.5 (86%)	132.54/142 (93.34%)	Total score (%)	136.29/158.5 (86%)	132.54/142 (93.34%)

The limited sparing model doesn’t scale to 30Gy Rx because the Hippocampus D0.03cc 11.26Gy x1.5 =16.89Gy which violates 16Gy protocol guidelines.

Colors are included for reliability. Bold or shaded areas highlight the differences from the moderate sparing model.

### 30-Gy detailed scorecard analysis of HMS-EC-WB and HSWBv2: expressing intent precisely

**Figure 2:**
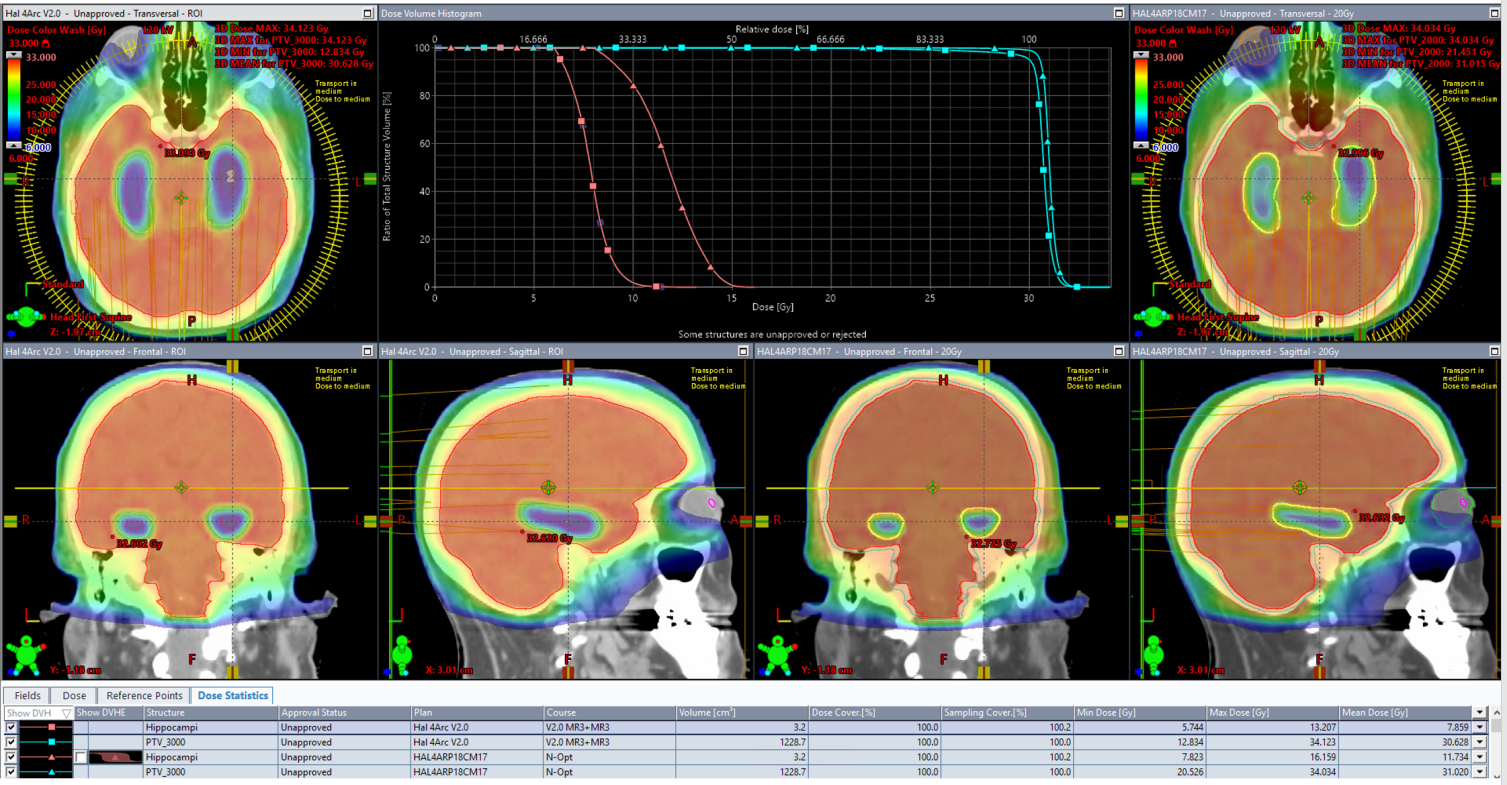
The DVH and isodose colorwash for qualitative analysis at 30Gy are seen on plans produced by each model on a representative validation case (patient 36).

**Table 4** shows the results of scores from both scorecards on plans created by both models. As expected, while both plans meet all protocol goals from NRG-CC001 and CCTG-CE.7, they both produce plans tailored to a differing, more specific, intent. Success in driving toward each specific intent is reflected by an increased share of the total possible score matching that scorecard’s associated RapidPlan model. The DVH and isodose colorwash for qualitative analysis at 30 Gy are seen on plans produced by each model on a representative validation case (patient 36; **Figure 2**).

**Table 4:** 30Gy detailed scorecard analysis of HMS-EC-WB & HSWBv2 with results of scores from both scorecards on plans created by both models.

Plan Score Comparison 30Gy								
RP model:	V2.0 Scorecard (142 points)				HMS-EC-WB Scorecard (158.5 points)			
	HSWBv2		HMS-EC-WB		HSWBv2		HMS-EC-WB	
patient 36	132.08	93.01%	111.06	78.21%	125.18	78.98%	131.85	83.19%
patient 37	133.24	93.83%	112.64	79.32%	127.46	80.42%	140.76	88.81%
patient 39	132.17	93.08%	115.12	81.07%	123.15	77.70%	137.85	86.97%
patient 40	133.39	93.94%	115.53	81.36%	123.35	77.82%	136.94	86.40%
patient 41	131.82	92.83%	118.49	83.44%	123.42	77.87%	134.07	84.59%
**average**	**132.54**	**93.34%**	**114.568**	**80.68%**	**124.512**	**78.56%**	**136.294**	**85.99%**

Colors are included for reliability. Bold or shaded areas highlight the differences from the moderate sparing model.

### 20-Gy detailed scorecard analysis of HLS-EC-WB, HMS-EC-WB, and HSWBv2: expressing intent precisely

**Figure 3:**
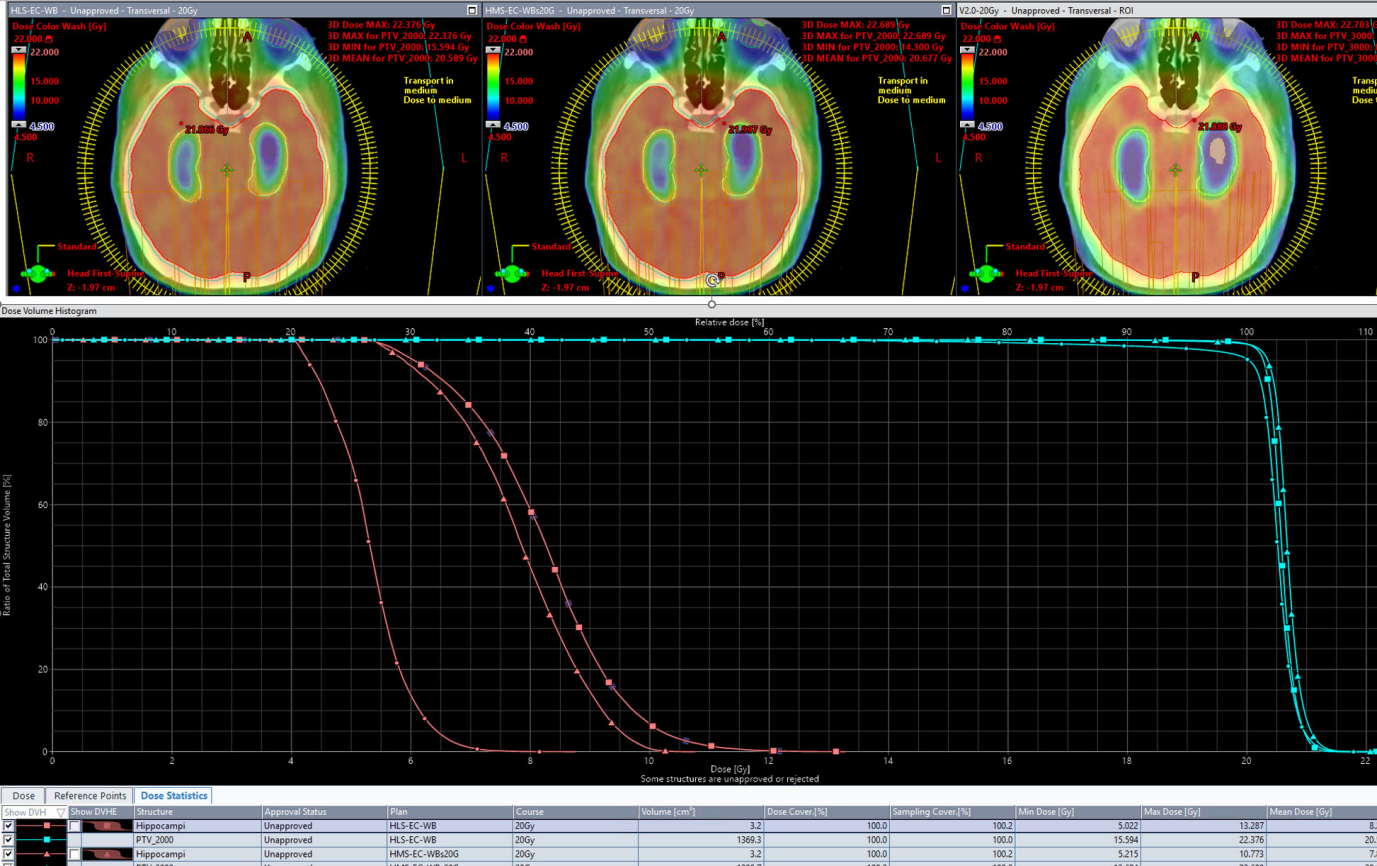
The DVH and isodose colorwash for qualitative analysis at 20Gy are seen on plans produced by each model on a representative validation case (patient 36).

**Table 5** shows the results of scores from all scorecards on plans created by all three models. Each RapidPlan™ model produces plans tailored to differing specific intent. Success in driving toward each specific intent is reflected by an increased share of the total possible score matching with that scorecard’s associated RapidPlan™ model. The DVH and isodose colorwash for qualitative analysis at 20 Gy are seen on plans produced by each model on a representative validation case (patient 36; **Figure 3**).

**Table 5:** 20Gy detailed scorecard analysis of HLS-EC-WB, HMS-EC-WB & HSWBv2 with results of scores from all scorecards on plans created by all three models.

Plan Score Comparison 20Gy (V2.0 and Moderate scorecard and plan doses scaled *.667)																		
Model	HLS-EC-WB Scorecard (158.5 points)						HMS-EC-WB Scorecard (158.5 points)						V2.0 Scorecard (142 points)					
	HSWBv2		HMS-EC-WB		HLS-EC-WB		HSWBv2		HMS-EC-WB		HLS-EC-WB		HSWBv2		HMS-EC-WB		HLS-EC-WB	
patient 36	123.42	77.87%	135.43	85.44%	141.32	89.16%	125.18	78.98%	131.85	83.19%	124.67	78.66%	132.08	93.01%	111.06	78.21%	110.48	77.80%
patient 37	128.49	81.07%	139.63	88.09%	143.93	90.81%	127.46	80.42%	140.76	88.81%	119.22	75.22%	133.24	93.83%	112.64	79.32%	112.35	79.12%
patient 39	116.72	73.64%	134.61	84.93%	143.31	90.42%	123.15	77.70%	137.85	86.97%	133.58	84.28%	132.17	93.08%	115.12	81.07%	117.58	82.80%
patient 40	114.66	72.34%	129.7	81.83%	137.3	86.62%	123.35	77.82%	136.94	86.40%	120.13	75.79%	133.39	93.94%	115.53	81.36%	116.68	82.17%
patient 41	123.76	78.08%	130.27	82.19%	141.54	89.30%	123.42	77.87%	134.07	84.59%	121.98	76.96%	131.82	92.83%	118.49	83.44%	112.31	79.09%
**Average**	**121.41**	**76.60%**	**133.928**	**84.50%**	**141.48**	**89.26%**	**124.512**	**78.56%**	**136.294**	**85.99%**	**123.916**	**78.18%**	**132.54**	**93.34%**	**114.568**	**80.68%**	**113.88**	**80.20%**

Colors are included for reliability. Bold or shaded areas highlight the differences from the moderate sparing model.

### HLS-EC-WB and HMS-EC-WB performance validation on TrueBeam

Both Enhanced Coverage models had their performance validated and scores reported in **Tables 6** and **7**.

**Table 6:** Limited Spring model (HLS-EC-WB) performance validated on TrueBeam and scores reported. When present, each asterisk represents a dosimetric scorecard metric that failed (scored zero points).

HLS-EC-WB	Halcyon	TrueBeam			
Patient	4 Arcs (Coplanar)	4 Arcs (Non-Coplanar)	3 Arcs (Coplanar)	4 Arcs (Coplanar)	HyperArc (Non-Coplanar)
36	141.32	137.2	128.01	137	137.88
37	143.93	141.02	140	142.28	138.16
39	143.31	137.32	127.29*	138.12	137.01
40	137.3	131.18*	117.89**	125.68*	131.36
41	141.54	131.07	132.13	135.85	133.11
**Average**	**141.48**	**136.65**	**133.38**	**138.31**	**135.50**

Colors are included for reliability. Bold or shaded areas highlight the differences from the moderate sparing model.

**Table 7:** Moderate Sparing model (HMS-EC-WB) performance validated on TrueBeam and scores reported.

HMS-EC-WB	Halcyon	TrueBeam			
Patient	4 Arcs (Coplanar)	4 Arcs (Non-Coplanar)	3 Arcs (Coplanar)	4 Arcs (Coplanar)	HyperArc (Non-Coplanar)
36	131.85	134.09	125.96	124.73	132.19
37	140.76	134.81	131.33	132.92	131.66
39	137.85	135.40	129.64	130.98	134.99
40	136.94	133.87	126.48	126.55	133.04
41	134.07	134.00	126.96	126.81	131.35
**Average**	**136.29**	**134.43**	**128.07**	**128.40**	**132.65**

Colors are included for reliability. Bold or shaded areas highlight the differences from the moderate sparing model.

**Figure 4:**
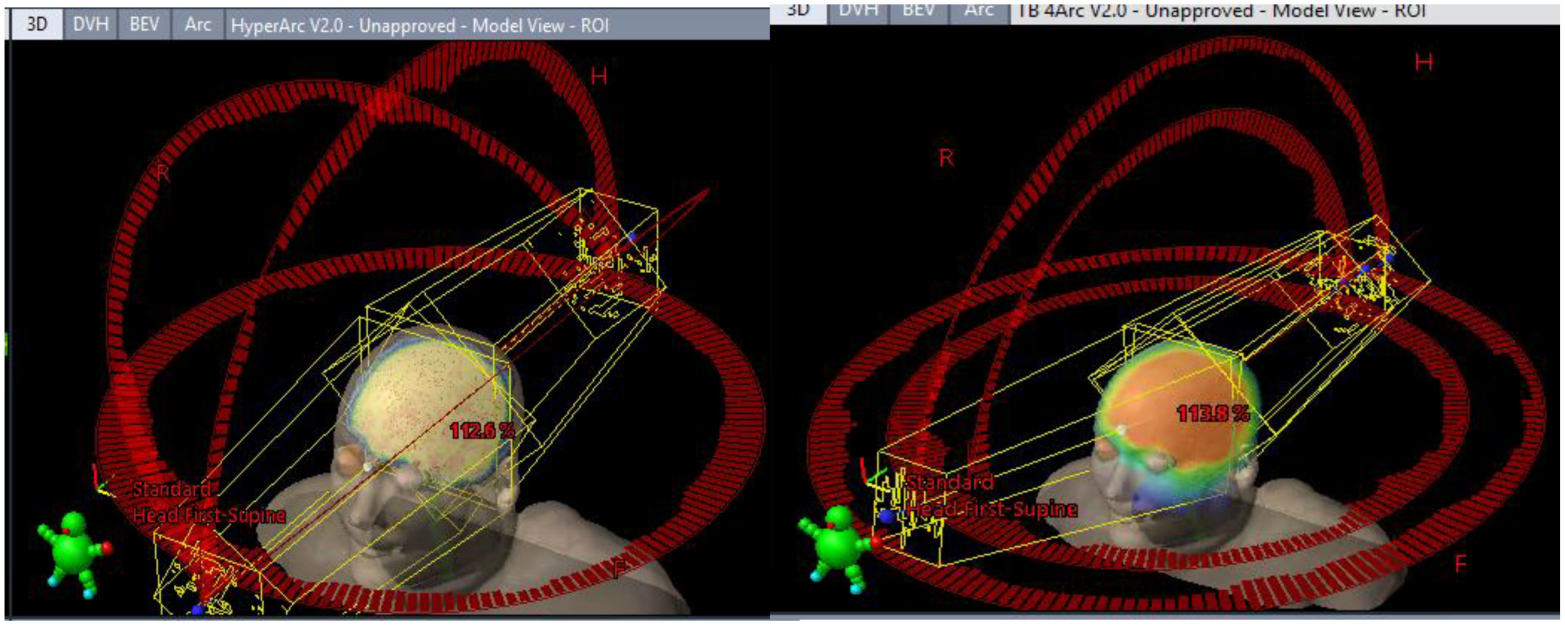
Differing TrueBeam noncoplanar arrangements. Automated full HyperArc left and non coplanar geometry right (4 Arcs Non-Coplanar: 2 full arcs 0° couch 315°/45° collimators and two vertex 180° (PA) -> 5° (from AP) 90° couch CW/CCW paired arcs with 315°/45° collimator).

Halcyon coplanar performance is unmatched due in part to the staggered dual-layer MLC design (0.01% 6xFFF nominal transmission for both banks vs. 1.36% M120) and faster leaf travel (5 vs. 2.5 cm/s M120) speed with complete overtravel capability that can better spare the hippocampi. However, when leveraging noncoplanar arc geometries, the performance gap shrinks (**Figure 4**). More details on the TrueBeam performance of each model (along with other treatment variables) can be found in the annex of the clinical description document attached to each RapidPlan™ model.

## Discussion

### Origin of the enhanced coverage models

These alternative hippocampal-sparing models were created as a result of exploring why a clinician might not use the 2022 HSWBv2 RapidPlan™ model. Authors of NRG-CC001 prioritize hippocampal sparing and expect it to be maximized while maintaining 95% coverage to the brain outside the hippocampal avoidance zone. HSWBv2 is an excellent choice whose single-click result is crafted to the intent of NRG-CC001 authors. However, satisfactory results are only likely when following the instructions on HSWBv2’s clinical description (i.e., Convergence Mode = Extended in Calculation Options and manually configuring the Minimum MU objective in optimization to a higher value forcing the required additional modulation to maximize dosimetric performance). As noted, some prefer to focus on homogeneity and maximize target coverage instead of hippocampal sparing. Physicians might aim for just under the maximum allowed D100% (minimum point dose in the hippocampus), which is listed as 9 Gy in both popular 30-Gy protocols (aiming for D100% = 8.8–8.9 Gy). Both these models aggressively push the hippocampus D100% As Low As Reasonably Achievable (ALARA) and average nearly 7 Gy instead of 9 Gy when prescribing 30 Gy to the brain while maintaining 99% target coverage and excellent dose homogeneity.

It was not possible to modify the existing HSWBv2 model for such a result, so new scorecards were developed (**Supplementary S1, S2**) to capture this differing intent and guide the creation of new RapidPlan™ models to best realize that intent. The limited-sparing scorecard was designed for an institution-specific 20-Gy prescription where homogeneity was prioritized before conformality, and relative maximum dose (D0.03cc) to the hippocampus was less constrained than in the popular 30-Gy protocols. The moderate-sparing scorecard attempted to take the best from all intents. It improved conformality, while still driving toward 99% prescription coverage of the brain but further prioritizing not only the minimum dose in the hippocampus but also the maximum (D0.03cc) dose to always achieve 30-Gy Rx D0.03 hippocampal constraint of 16 Gy (HMS-EC-WB D0.03cc = 14.4 Gy in the five-case validation set). Most clinicians will likely favor the HSWBv2 or the moderate-sparing models, which scale to any prescription—the minor improvement in target homogeneity at the expense of a higher maximum dose to the hippocampus of the limited-sparing model is only recommended for those sure they are comfortable with such a tradeoff and are only treating to 20 Gy.

### Limitations

CCTG-CE.7 allows for partial hippocampal sparing in cases where metastases overlap with hippocampal avoidance zone (**Supplementary S6**). Training and validation cases for this work did not include metastasis structures. Therefore, these models are only intended to be used directly on cases with no gross nearby disease nearby, or where gross disease is nearby and potentially un-contoured, not where gross disease is inside the hippocampal avoidance zone where metastasis contouring would be required for partial hippocampal avoidance. Furthermore, none of the RapidPlan™ models described in this work have been designed for SIB. However, the clinical description document with each model includes a single patient example with suggested structure margins when utilizing these models with cases where metastases are contoured in the hippocampal avoidance zone, with or without SIB treatment (**Supplementary S7**). Future study is needed with a larger number of cases to assess treatment plan quality with SIB prescription using these models.

While different models were not created for each intent and possible prescription dose level combination, two scalable models featured in this work (moderate sparing and HSWBv2) have been demonstrated at both 30 Gy in 10 fractions and 20 Gy in 5 fractions. Outside the scope of this work, either scalable model could be an excellent candidate to test with alternate prescriptions (25 Gy in 10 fractions/NRG CC003).

These limited and moderate-sparing models were validated on only five cases and did not include statistical analysis. However, HSWBv2 model work done in 2022 utilized the same CT and structures for both the training and validation sets as this work ensuring no geometric outliers, and all cases were replanned, as described, precluding dosimetric outliers. This limitation in the number of training and validation cases can be considered in the context of anecdotal positive feedback from multiple institutions implementing HSWBv2 clinically. The 2022 HSWBv2 “how far can we reduce the dose?” work is highly sited, features similar methods, and also lacks statistical analysis (2). This work demonstrating the limited- and moderate-sparing models does not include direct dosimetric comparison with compatible intent limited and moderate sparing, clinically delivered, treatment plans.

### Automation possibilities for various intents

A variety of hippocampal-sparing intents are covered by this work, and free, public models are available, which maximize each intent. Significant effort savings are likely possible when a treatment planner has Eclipse and a RapidPlan™ license, is making a VMAT hippocampal sparing plan with Halcyon or TrueBeam, and uses one of these models. Regarding dosimetric plan quality, the authors of this work are confident that plans produced by these models will be dosimetrically superior to most manual attempts. Clinicians are invited to download the MAAS-PlanScoreCard tool, the associated scorecard, which reflects their desired clinical intent and its associated RapidPlan™ model. Then, compare a plan created manually (or through other methods) and see if it scores higher than the plan generated by one of these RapidPlan™ models without user interaction.

### The future of dosimetric scorecards

This work has demonstrated the power of using dosimetric scorecards to tune the automatically generated optimization objective priorities embedded in RapidPlan™ models. Dosimetric scorecards can be used in several other ways. They can be used in the clinic to retrospectively or prospectively capture a clinical intent, which can drive plan quality and consistency in manual planning, even for those with less clinical treatment planning experience (10). Alternatively, they can be used to evaluate dosimetric improvement of varying the number of static gantry IMRT fields when establishing a new technique (11) or used to help tune an Ethos Clinical Directive template (12). All future published dosimetric protocols could not only include the pass/variation/fail dose constraints but also additionally include a dosimetric scorecard to comprehensively articulate the full dosimetric intent with precision. Capturing such precision is not possible with pass/variation/fail dose constraints or simple ranked priorities. This work demonstrates various treatment plans, all of which achieve the base protocol dose constraints, but can vary wildly between maximizing prescription coverage or sparing. When published protocol goals are loose and specific clinical intent is not well articulated, multiple legitimate seeming interpretations of the intent are possible. As a field, radiation oncology accepting this level of variation in supposedly protocol enforced intent is troubling. Variations in intent cause variations in care adding needlessly to noise in outcome data in published studies.

In a future where precise dosimetric scorecards are crafted at the protocol level, published and popularized by future protocol authors, treatment planning dose optimizers could be constructed to accept direct piecewise linear DVH value scorecards as their exclusive input. Then, the system could simply give the user the best scoring plan as defined by the scorecard for a given beam set and machine. The amount work required to create highly tuned RapidPlan™ models is extremely laborious as described in this document. Dose optimizers that could directly output the best version of a precise clinical intent would be highly preferable.

## Conclusion

There are now three high-quality fully automated treatment plan-generating RapidPlan™ models for hippocampal-sparing whole brain, each developed with a distinct dosimetric scorecard to articulate precise intent. The new alternate Hippocampal (Limited/Moderate)-Sparing Enhanced-Coverage Whole-Brain RapidPlan™ models (HLS-EC-WB and HMS-EC-WB) are largely tailored toward an increase in PTV Rx coverage (98%–99%) at some expense to hippocampal sparing. All three RapidPlan™ models (13), for example, DICOM plans, associated dosimetric scorecards, as well as links to the GitHub repository for MAAS-PlanScorecard tool (9), are publicly available.

## Data Availability

The datasets presented in this study can be found in online repositories. The names of the repository/repositories and accession number(s) can be found below: https://medicalaffairs.varian.com/wholebrain-moderate-hippocampalsparing-30gy-vmat2.
