# Supplementary material for: Dosimetric scorecards express precise clinical intent: alternate hippocampal-sparing whole-brain RapidPlan models favoring target coverage and homogeneity at 30 and 20 Gy
Source: Front Oncol. 2024 Nov 27;14:1465171. doi: 10.3389/fonc.2024.1465171 (PMC11632126; doi:10.3389/fonc.2024.1465171)
Supplement: Supplementary file 1 [file DataSheet1.docx]

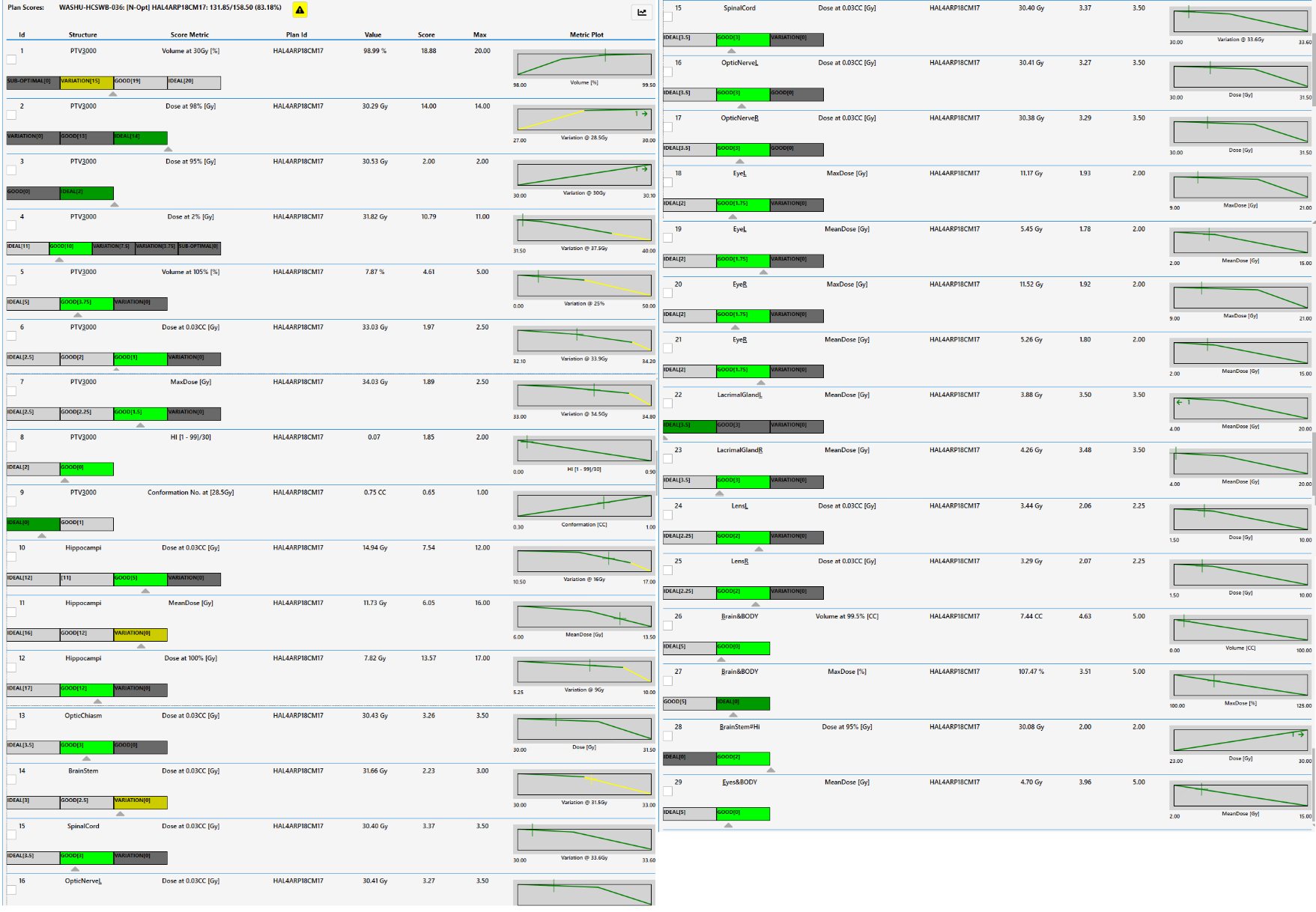


Supplementary **Figure S1**- New scorecards were created to capture different hippocampal sparing intent, previous HSWBv2 scorecard shown here.


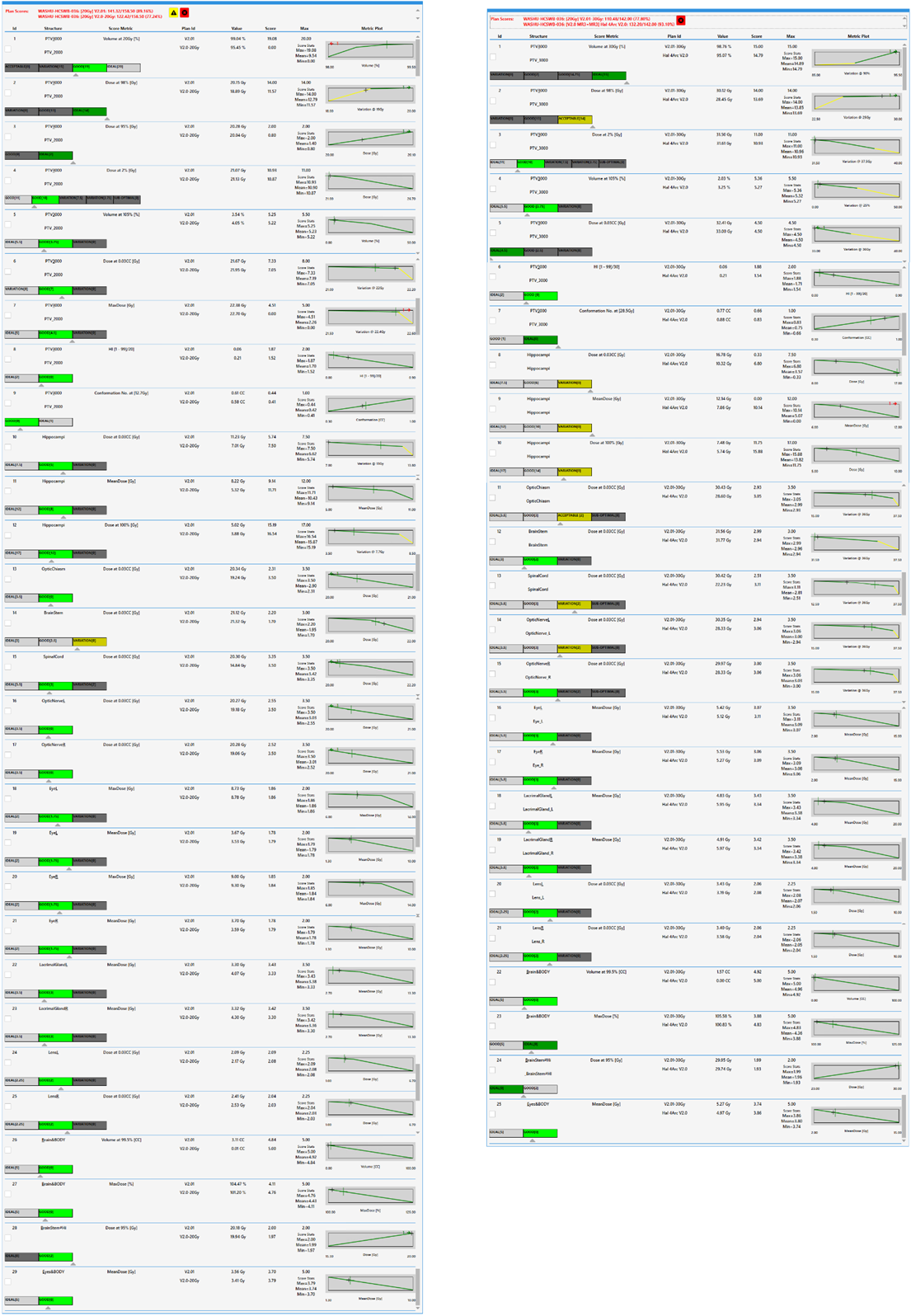


Supplementary **Figures 2a & 2b** : New scorecards were created to capture different hippocampal sparing intent.

|  | **V2.0 Scorecard=142** | **Limited Sparing Scorecard = 158.5 Total points** | |  |
| --- | --- | --- | --- | --- |
|  | **Final Result** | **created by modV2.0 model** | **created by initial HLS-EC-WB** | **Final Model Result** |
| **Patient** | **Final V2.0 Model** | **Training Set for initial HLS-EC-WB** | **Training Set for final HLS-EC-WB** | **Final HLS-EC-WB** |
| Patient 1 | 132.35 | 141.86 | 145.71 | 145.44 |
| Patient 3 | 132.47 | 145.2 | 144.86 | 143.44 |
| Patient 4 | 137.17 | 147.19 | 147.07 | 149.02 |
| Patient 5 | 129.81 | 142.07 | 147.21 | 147.2 |
| Patient 8 | 132.53 | 140.78 | 143.11 | 142.5 |
| Patient 9 | 132.6 | 140.45 | 141.33 | 142.38 |
| Patient 11 | 132.32 | 133.87 | 138.56 | 140.29 |
| Patient 13 | 131.92 | 144.49 | 145.4 | 145 |
| Patient 16 | 136.6 | 141.81 | 143.81 | 146.68 |
| Patient 19 | 134.64 | 139.96 | 138.36 | 142.39 |
| Patient 20 | 131.66 | 135.18 | 140.46 | 140.36 |
| Patient 21 | 131.36 | 133.19 | 134.17 | 139.98 |
| Patient 23 | 134.14 | 145.68 | 146.29 | 144.43 |
| Patient 24 | 133.82 | 137.54 | 143.45 | 141.3 |
| Patient 25 | 134.88 | 145.97 | 146.43 | 146.63 |
| Patient 27 | 133.96 | 146.26 | 146.81 | 145.79 |
| Patient 28 | 131.32 | 139.94 | 141.11 | 140.14 |
| Patient 30 | 133.23 | 140.83 | 140 | 143.21 |
| Patient 34 | 131.16 | 141.55 | 143.28 | 143.15 |
| Patient 35 | 132.72 | 144.64 | 145.4 | 145.29 |
| Patient 44 | 129.91 | 137.45 | 135.43 | 140.17 |
| Patient 45 | 129.64 | 139.56 | 141.95 | 143.56 |
| Patient 47 | 133.63 | 145.21 | 145.85 | 146.5 |
| Patient 48 | 135.3 | 145.59 | 146.16 | 146.85 |
| Patient 49 | 134.7 | 144.78 | 146.3 | 145.5 |
| Patient 50 | 131.7 | 144.62 | 144.8 | 144.11 |
| Patient 52 | 132.01 | 138.99 | 142.36 | 141.31 |
| Patient 54 | 134.41 | 145.38 | 143.13 | 145.87 |
| Patient 55 | 132.49 | 135.02 | 135.9 | 140 |
| Patient 57 | 134.69 | 140.16 | 140.75 | 145.58 |
| Patient 60 | 131.74 | 134.59 | 131.7 | 135.36 |
| Patient 64 | 134.42 | 141.49 | 146.32 | 145.41 |
| Patient 65 | 133.45 | 143.75 | 143.68 | 143.29 |
| Patient 66 | 132.58 | 136.49 | 138.8 | 140.33 |
| Patient 68 | 132.24 | 134.1 | 143.46 | 144.42 |
| Patient 69 | 130.8 | 131.62 | 126.21 | 135.51 |
| Patient 70 | 133.5 | 135.17 | 139.71 | 140.47 |
| Patient 71 | 132.54 | 141.31 | 143.41 | 143.58 |
| Patient 72 | 132.72 | 140.79 | 138.28 | 142.04 |
| Patient 77 | 131.67 | 142.35 | 144.01 | 142.5 |
| Patient 80 | 134.76 | 147.27 | 147.11 | 146.36 |
| Patient 85 | 135.24 | 144.27 | 140.82 | 145.73 |
| **Average** | **132.9714286** | **140.9147619** | **142.1180952** | **143.3111905** |

Supplementary **Table S1**: The initial plans created from HSWBv2.0 model were scaled to 20Gy and replanned with offset hippocampal sparing objectives, those cases became the training set for the initial Limited sparing model. The initial limited sparing model was used to generate plans that were used to train the final limited sparing model. This so-called recursive process was used to ensure that the final limited sparing model’s training set only consisted of plans generated from the initial limited sparing model’s output. The plan scores were evaluated at each step of the process, and the model’s optimization objective set was adjusted multiple times based on those evaluations.

|  | **V2.0 Scorecard = 142** | **HMS-EC-WB Scorecard = 158.5 Total points** | |
| --- | --- | --- | --- |
|  | **Final Result** | **created by modV2.0 model with offset objectives** | **Final Result** |
| **Patient** | **Final V2.0 Model** | **Training Set for initial HMS-EC-WB** | **Final HMS-EC-WB Model** |
| Patient 1 | 132.35 | 137.17 | 140.14 |
| Patient 3 | 132.47 | 136.37 | 134.52 |
| Patient 4 | 137.17 | 146 | 146.26 |
| Patient 5 | 129.81 | 140.69 | 141.48 |
| Patient 8 | 132.53 | 136.73 | 136.49 |
| Patient 9 | 132.6 | 135.36 | 135.6 |
| Patient 11 | 132.32 | 131.73 | 133.95 |
| Patient 13 | 131.92 | 135.29 | 140.56 |
| Patient 16 | 136.6 | 146.44 | 146.22 |
| Patient 19 | 134.64 | 139.24 | 138.92 |
| Patient 20 | 131.66 | 132.07 | 132.13 |
| Patient 21 | 131.36 | 133.81 | 134.26 |
| Patient 23 | 134.14 | 139.89 | 140.72 |
| Patient 24 | 133.82 | 141.03 | 141.03 |
| Patient 25 | 134.88 | 139.53 | 137.89 |
| Patient 27 | 133.96 | 141.26 | 141.62 |
| Patient 28 | 131.32 | 133.53 | 131.95 |
| Patient 30 | 133.23 | 138.34 | 140.85 |
| Patient 34 | 131.16 | 135.78 | 136.87 |
| Patient 35 | 132.72 | 135.71 | 136.14 |
| Patient 44 | 129.91 | 134.27 | 125.48 |
| Patient 45 | 129.64 | 132.47 | 134.31 |
| Patient 47 | 133.63 | 141.32 | 142.91 |
| Patient 48 | 135.3 | 143.18 | 143.28 |
| Patient 49 | 134.7 | 143.56 | 144.12 |
| Patient 50 | 131.7 | 137.23 | 138.78 |
| Patient 52 | 132.01 | 134.59 | 136.65 |
| Patient 54 | 134.41 | 140.28 | 141.77 |
| Patient 55 | 132.49 | 133.25 | 133.47 |
| Patient 57 | 134.69 | 139.7 | 140.43 |
| Patient 60 | 131.74 | 133.29 | 133.25 |
| Patient 64 | 134.42 | 138.93 | 139.33 |
| Patient 65 | 133.45 | 136 | 139.27 |
| Patient 66 | 132.58 | 130.75 | 131.95 |
| Patient 68 | 132.24 | 138.88 | 138.64 |
| Patient 69 | 130.8 | 124.41 | 126.85 |
| Patient 70 | 133.5 | 134.59 | 134.95 |
| Patient 71 | 132.54 | 135.08 | 134.27 |
| Patient 72 | 132.72 | 135.85 | 135.08 |
| Patient 77 | 131.67 | 135.78 | 137.46 |
| Patient 80 | 134.76 | 141.45 | 143.19 |
| Patient 85 | 135.24 | 140.6 | 140.3 |
| **Average** | 132.9714286 | 137.1769048 | 137.70 |

Supplementary **Table S2**: Dosimetric scorecard results throughout the creation process for moderate sparing model. As a reference, the column on the left shows scores achieved by HSWBv2 scorecard and model. The middle column are cases scored with moderate sparing scorecard and created with HSWBv2 model with offset objectives, these became the training set for the initial model. Unfortunately, plans created with the initial model overall scored worse but had more aggressive hippocampal sparing (scores for the plans in this step are not shown). However, those cases with extra aggressive hippocampal sparing became the training set for the final moderate model, results of which are shown in the right column, finally outscoring the plans created by the previous HSWBv2 model with simple offset objectives.


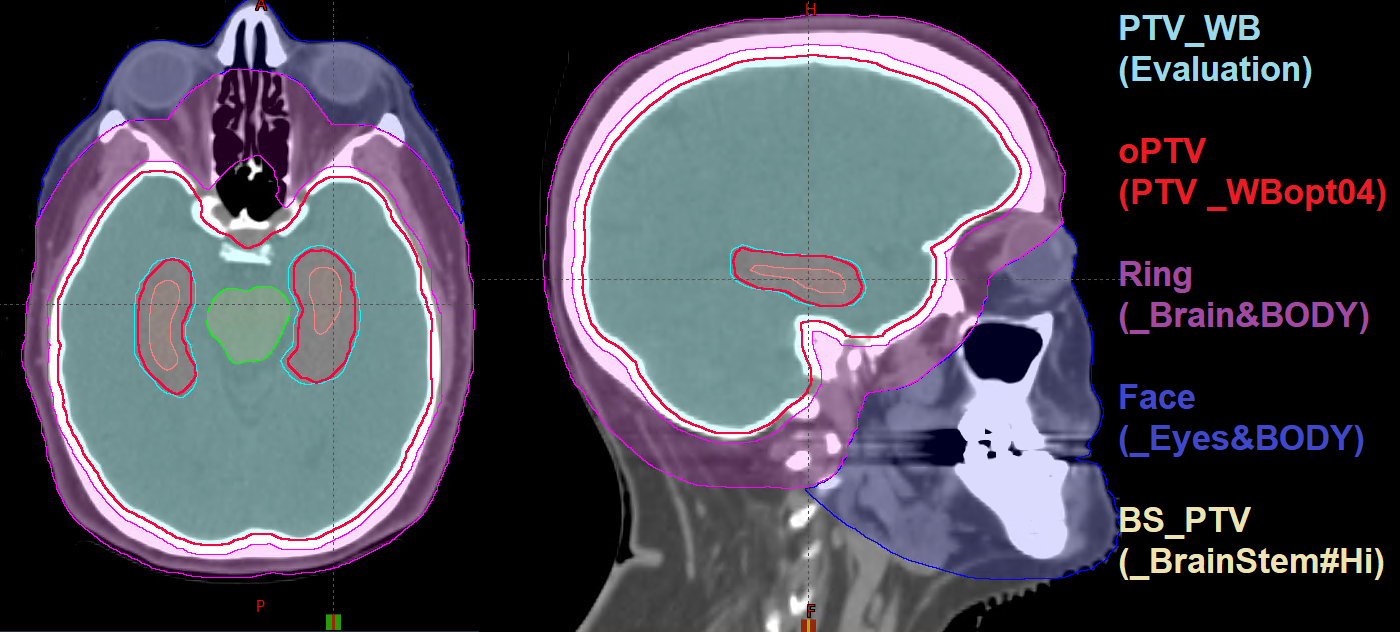


Supplementary **Figure S3**: Optimization structures needed for Enhanced Coverage models. Can be created automatically with MAAS-PlanScorecard tool. PTV_WBopt04 extends inside hippocampal avoidance zone.


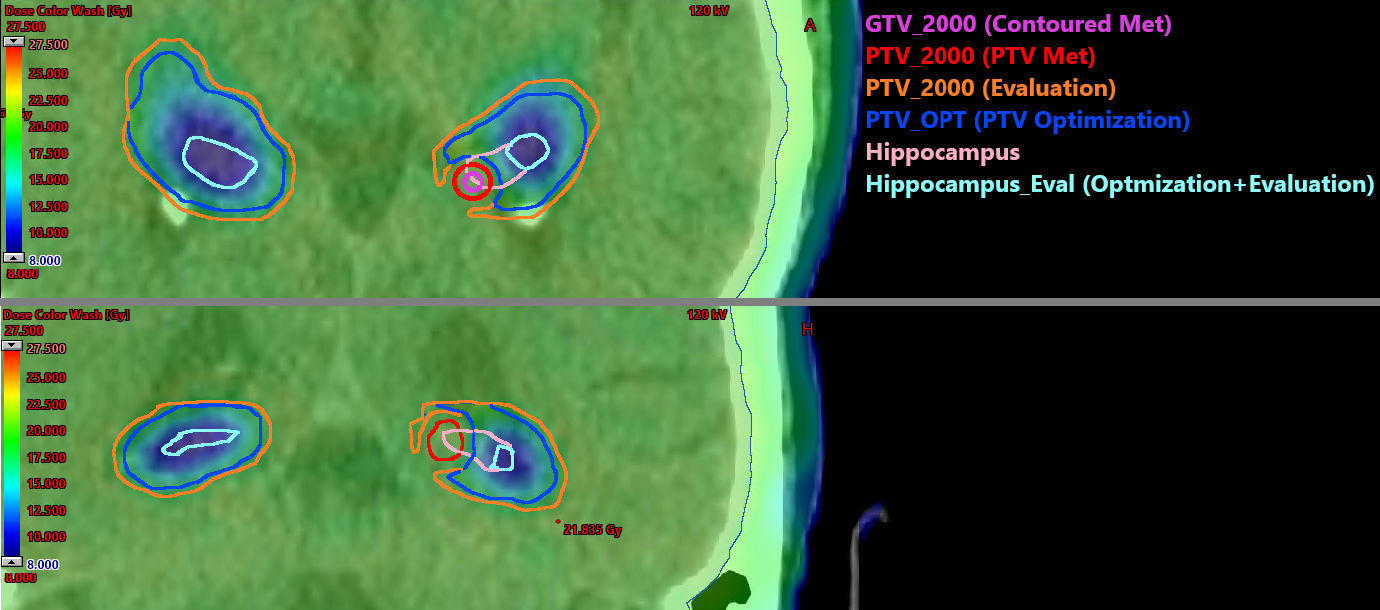


Supplementary **Figure S4**: CCTG CE. 7 allows for partial hippocampal sparing wherever possible even with metastasis close to or overlapping the hippocampus. All metastasis within 5mm of hippocampus are to be segmented per CCTG CE. 7.


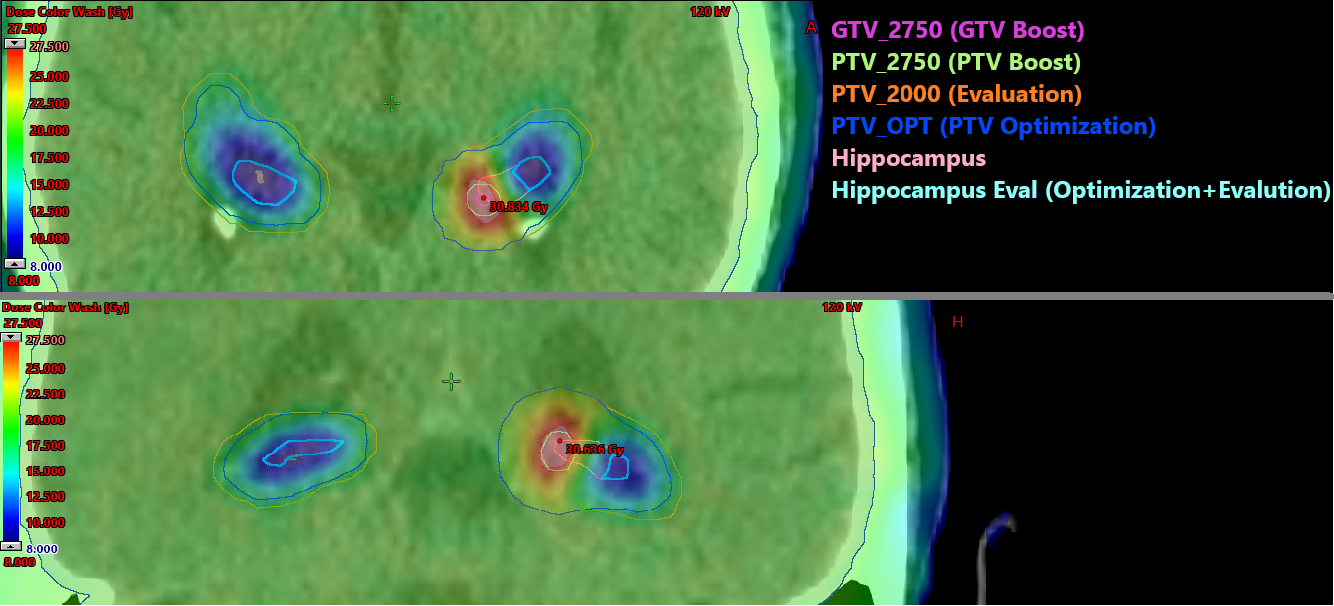


Supplementary **Figure S5**: The clinical description document included with each model includes a single patient example with suggested structure margins when utilizing any of these models for Simultaneous Integrated Boost (SIB) treatment
